# DualPlaqueNet with dual-branch structure and attention mechanism for carotid plaque semantic segmentation and size prediction

**DOI:** 10.3389/fphys.2025.1629637

**Published:** 2025-07-15

**Authors:** Lili Deng, Xingyu Duan, Yongxiang Sun, Yunling Wang, Dongmei Song, Xiaokai Duan

**Affiliations:** ^1^ Department of General Medicine, The First People’s Hospital of Zhengzhou, Zhengzhou, Henan, China; ^2^ First Clinical Medical College, Ningxia Medical University, Yinchuan, Ningxia, China; ^3^ Department of Ultrasound, The First People’s Hospital of Zhengzhou, Zhengzhou, Henan, China

**Keywords:** carotid plaque, semantic segmentation, carotid ultrasound, image analysis, deep learning

## Abstract

**Background:**

With global aging and lifestyle changes, carotid atherosclerotic plaques are a major cause of cerebrovascular disease and ischemic stroke. However, ultrasound images suffer from high noise, low contrast, and blurred edges, making it difficult for traditional image processing methods to accurately extract plaque information.

**Objective:**

To establish a deep learning-based DualPlaqueNet model for semantic segmentation and size prediction of plaques in carotid ultrasound images, thereby providing comprehensive and accurate auxiliary information for clinical risk assessment and personalized diagnosis and treatment.

**Methods:**

DualPlaqueNet uses a dual-branch architecture combined with attention mechanisms and joint loss functions to optimize segmentation and regression. Notably, a multi-layer one-dimensional convolutional structure is introduced within the Efficient Channel Attention (ECA) module. The original dataset contained 287 carotid ultrasound images from patients at Zhengzhou First People’s Hospital, which were divided into training, validation, and test sets. Model training, validation, and testing were performed after preprocessing and data augmentation of the training set. Its performance was compared with three other models.

**Results:**

In the plaque semantic segmentation task, DualPlaqueNet outperformed the other three models across all metrics, achieving MIoU of 88.91 ± 1.027 (%), IoU (excluding background) of 88.22 ± 1.065 (%), DSC of 89.95 ± 1.102 (%), and Accuracy of 95.98 ± 0.073 (%). For plaque size prediction, this model demonstrated lower MSE and MAE, along with a higher coefficient of determination *R* ^2^, proving its ability to accurately extract plaque size information from ultrasound images.

**Conclusion:**

The dual-branch design and attention mechanisms of DualPlaqueNet effectively address the challenges of ultrasound images, achieving precise segmentation and size prediction, demonstrating its potential as an auxiliary tool for future clinical applications.

## 1 Introduction

In recent years, as global aging accelerates and lifestyles change, cardiovascular diseases have gradually emerged as a major public health threat (Azeez, 2023; Bayoumi and Karasik, 2021; Goldsborough et al., 2022). The formation and progression of carotid atherosclerotic plaques are considered to be key pathological foundations for cerebrovascular diseases and ischemic stroke, and their accurate detection and quantitative analysis are of great significance for clinical prevention, risk assessment, and treatment decision-making (Ihle-Hansen et al., 2023; Miao et al., 2022). Carotid plaques not only reflect the severity of systemic arteriosclerosis but also provide individualized health management recommendations for patients (Hou et al., 2024; van Dam-Nolen et al., 2022). Currently, the diagnosis of carotid plaques requires ultrasound examinations. However, ultrasound physicians must spend long hours in front of display screens, which can lead to occupational ailments such as eye strain and back pain. Moreover, diagnoses made by different sonographers are prone to subjective errors due to varying levels of clinical expertise, and even the same physician may demonstrate different diagnostic efficiency depending on their level of fatigue. Therefore, automatically and accurately segmenting plaque regions from ultrasound images while predicting plaque size and improving efficiency has become a critical issue that urgently needs to be addressed in the field of carotid ultrasound image analysis.

Ultrasound imaging, due to its non-invasive, real-time, cost-effective, and widely applicable nature, has been extensively used for the clinical detection of carotid plaques. However, inherent limitations of ultrasound images—such as high noise levels, low contrast, and blurred edges—make traditional image processing algorithms prone to interference when segmenting plaques, and they struggle to capture the subtle morphological features of plaques (Luo et al., 2021; Singh et al., 2023). Specifically, the main challenges in ultrasound image analysis of carotid plaques are: (1) intense speckle noise and echo attenuation result in very low contrast between the plaque and surrounding tissue; (2) calcified plaques produce strong shadowing effects, causing fragmented boundaries and distorted morphology; (3) considerable variability in vessel anatomy and plaque types across patients makes model generalization difficult; and (4) probe motion and arterial pulsation introduce dynamic artifacts, further degrading segmentation accuracy. In recent years, deep learning techniques, particularly convolutional neural networks (CNNs), have achieved remarkable success in medical image segmentation, greatly advancing the automation of medical image analysis. At present, in addition to the research on cardiac and breast ultrasound, many scholars are also focusing on carotid plaque image segmentation, as shown in Table 1; (Huang et al., 2023). For instance, Zhou et al. proposed a deep - learning - based method for automatically measuring the total plaque area in B- mode ultrasound images. Trained on a small dataset with the UNet++ integrated algorithm, it can efficiently and accurately measure the total plaque area (TPA) and has shown good generalization ability on datasets acquired from different devices (Zhou et al., 2021).

**TABLE 1:** Relevant DL-based plaque segmentation techniques and their main features: investigators, references, publication year, segmentation techniques used, workflow type (semi-automatic or fully automatic), dataset size, data type (frames or video), plaque presence (“Yes” or “Not All”), performance metrics, and major advantages and disadvantages of the techniques.

Investigator	Year	Segmentation technique	Workflow	Dataset size	Image modality	Presence of Plaque	DSC	Merits and demerits
Meshram et al. (2020)	2020	Dilated U-Net	Semi-Automatic	352	Video	Yes	0.84	1. Better performance for complex cases. 2. Acceptable performances require sonographer input via a bounding box on plaque
Xie et al. (2020)	2020	Cascaded U-Net	Semi-Automatic	500	Frames	Yes	0.69	1. Without any pre-processing or narrowing the ROIs. 2. Segmentation of both the vessel and plaque. 3. Unacceptable performance
Mi et al. (2021)	2021	MBFF-Net	Fully-Automatic	430	Frames	Yes	0.78	1. Outperformed state-of-the-art methods. 2. Physiological characteristics. 3. Precise boundary detection. 4. Complex network structure
Jain et al. (2021)	2021	HDL networks	Fully-Automatic	970	Frames	Yes	0.895	1. Good robustness and scalability. 2. Acceptable speed. 3. Data argumentation. 4. Large memory size. 5. Poor reusability
Liapi et al. (2022)	2022	U-Net	Semi-Automatic	210	Video	Not All	0.736	1. Designed workflow for CAD system. 2. Intensity-normalized preprocessing. 3. Not well performance
Jain et al. (2022)	2022	HDL networks	Fully-Automatic	970	Frames	Yes	0.868	1. Faster training, small memory size. 2. Multiethnic database. 3. Outperform Autoencoder based model. 4. Only far wall plaque measurement

To address these issues, this paper proposes a novel multi-task joint learning model—DualPlaqueNet. The model adopts a dual-branch network architecture that is specifically designed for the tasks of plaque semantic segmentation and size prediction, and it achieves information sharing and collaborative optimization between the two tasks through a cross-fusion mechanism. Specifically, one branch of DualPlaqueNet is dedicated to extracting global semantic features to capture the overall morphology of plaques in complex backgrounds, while the other branch focuses on local detailed features to precisely delineate plaque edges and size information. By designing a joint loss function, the model is able to simultaneously optimize both segmentation and size prediction tasks during training, allowing these tasks to complement each other and collectively enhance the overall performance and robustness of the model.

Based on the research work of the DualPlaqueNet model, this paper aims to establish a multi-task joint optimization framework capable of performing both plaque semantic segmentation and size prediction simultaneously. This framework not only enhances the accuracy of plaque detection but also provides clinicians with richer and more intuitive diagnostic information, ultimately reducing the physicians’ workload.

## 2 Materials and methods

### 2.1 Data collection and grouping

In this study, a total of 523 patients underwent carotid ultrasound examination. Based on inclusion and exclusion criteria, 287 patients were ultimately selected, with one high-quality image (manually screened) chosen from each patient’s ultrasound images. These patients were from the outpatient and inpatient departments of Zhengzhou First People’s Hospital, and their carotid ultrasound images constituted the original image dataset for this study.

Inclusion Criteria: Patients who underwent carotid ultrasound examinations and were found to have carotid plaques.

Exclusion Criteria: (1) Patients whose ultrasound reports did not indicate the location of the plaques; (2) Patients whose ultrasound reports did not describe the long or short diameters of the plaques; (3) Patients who did not sign the informed consent form.

This study was conducted in accordance with the Declaration of Helsinki and received approval from the Hospital Ethics Committee (Ethics Review Committee of the First People’s Hospital of Zhengzhou, No. 2024-069). Prior to collecting the carotid ultrasound images, all participants or their guardians signed a consent form, ensuring the ethical compliance of the study.

Two physicians with 10 years of experience in ultrasound confirmed the plaque locations and sizes in the carotid ultrasound reports, and they manually annotated the plaques in the ultrasound images. In cases of disagreement, the two physicians consulted with a senior physician with over 25 years of clinical experience until consensus was reached.

To effectively train, optimize, and evaluate the model, the ultrasound image dataset was randomly divided into training, validation, and test sets at a ratio of 7:1:2, ensuring the scientific and reliable process of model training, validation, and evaluation.

### 2.2 Data preprocessing

**FIGURE 1:**
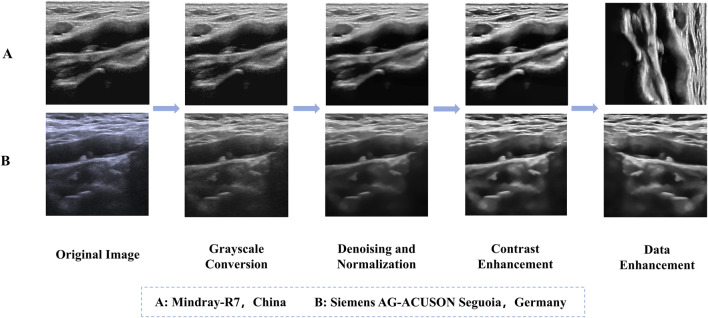
Example of the data preprocessing process. **(A)** Mindray-R7, China, **(B)** Siemens AG-ACUSON Seguoia, Germany.

Prior to preprocessing, patients’ personal information was removed from the ultrasound images to protect privacy. Our ultrasound brand involves two types, namely, Mindray-R7, China and Siemens AG-ACUSON Seguoia, Germany. For the convenience of subsequent analysis, the ultrasound images of these two brands were subjected to the same preprocessing steps to standardize them. Considering factors such as segmentation accuracy and training speed, the images were normalized and enhanced for contrast to improve detail representation. Additionally, to enhance the model’s generalizability and robustness, data augmentation was performed on the training set (Table 2). Specific augmentation techniques included elastic deformation, rotation, scaling, and flipping operations. These data augmentation methods not only effectively expanded the training set and prevented model overfitting, but also simulated different clinical scenarios and equipment variations, thereby improving the model’s adaptability in practical applications (Yan et al., 2024; Piao et al., 2022). Figure 1 illustrates an example of the data preprocessing process.

**TABLE 2:** Comparison of sample numbers before and after data augmentation.

Datasets	Training set (images)	Validation set (images)	Test set (images)
Pre-Augmentation	200	28	59
Post-Augmentation	1,600	28	59

### 2.3 Model construction

**FIGURE 2:**
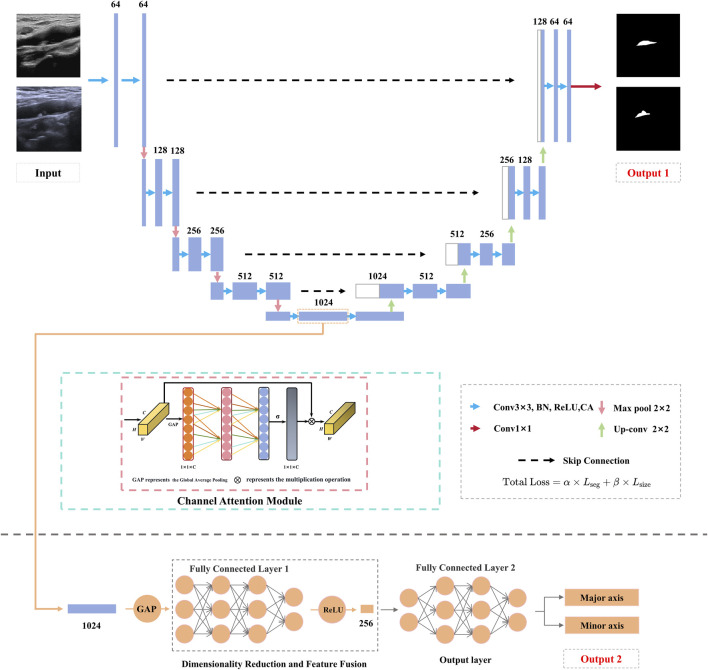
Schematic diagram of the DualPlaqueNet model architecture.

The proposed DualPlaqueNet model (Figure 2) introduces innovative improvements based on the traditional U-Net architecture, aiming to address the semantic segmentation of carotid plaque ultrasound images and the prediction of plaque size. The model first adopts the U-Net encoder-decoder structure (Tseng et al., 2023; Yi et al., 2023), extracting multi-scale features through down-sampling and integrating low-level details with high-level semantic information via up-sampling and skip connections. Additionally, an attention mechanism (Alshomrani et al., 2023; Sheng et al., 2022) is incorporated to achieve precise segmentation of plaque regions. In this study, we adopted and improved the Efficient Channel Attention (ECA) module to enhance the model’s performance in plaque region segmentation. Moreover, we deployed the ECA module at every feature extraction layer in the encoder. The ECA module generates channel weights through local cross-channel interaction, helping the network more precisely capture feature information from different channels, thereby improving segmentation performance. In the original ECA module, a single one-dimensional convolution layer was used to compute channel weights. We introduced a multi-layer one-dimensional convolution structure to extract feature information at different levels layer by layer, further optimizing the channel weight computation process and enhancing the model’s ability to capture complex image features.

Regarding the choice of ECA over other more advanced attention mechanisms, this is mainly due to its efficiency and low computational overhead. ECA uses one-dimensional convolution to compute channel weights, making it have lower computational complexity compared to other attention mechanisms (such as multi-head self-attention or Manhattan attention). When processing medical images, especially segmentation tasks for small targets like plaque regions, ECA can maintain efficient inference speed while effectively improving performance through relatively low computational overhead. Although mechanisms like multi-head self-attention and Manhattan attention can provide stronger feature capture capabilities, they typically have high computational overhead, especially when processing high-resolution medical images, which may lead to slower training and inference speeds. Therefore, selecting the ECA module can improve model performance while ensuring efficient computational efficiency.

Although measuring dimensions on plaque segmentation results is a feasible approach, this method may overlook the complexity of the dimension prediction task. Dimension prediction is not merely simple post-processing based on segmentation results; it involves comprehensive understanding of multiple factors such as plaque morphology, boundaries, and position. If the model relies solely on segmentation results for dimension measurement, it may ignore the detailed features of plaques, thus affecting the accuracy of dimension prediction. Through joint training, we enable the model to learn the low-level features and semantic information required for dimension prediction while performing plaque segmentation. This design allows the model to simultaneously optimize both tasks, capture the interconnections between them, and enhance the model’s comprehensive understanding of plaques. Therefore, DualPlaqueNet introduces a novel branch dedicated to plaque size prediction. This branch extracts plaque morphological information from the deeper features of the encoder and, through a series of convolutional and fully-connected layers, regresses the plaque’s long and short diameters. To enable multi-task collaborative learning, a joint loss function is employed, with an automatic parameter tuning method used to determine the values of parameters α and β, thus balancing the semantic segmentation loss and regression loss to promote mutual optimization between the two tasks. In this study, we used Cross-Entropy Loss and Mean Squared Error Loss (MSE Loss) as the loss functions for the two main tasks. We used α and β to control the relative importance of the segmentation task and the size prediction task. See Equations 1, 2 for details.

$$L_{loss}=\alpha \times L_{seg}+\beta \times L_{size}$$ (1)

$$L_{loss}=\alpha \left[-\sum_{i=1}^{N}\left[y_{i}\log\left(p_{i}\right)+\left(1-y_{i}\right)\log\left(1-p_{i}\right)\right]\right]+\beta \left[\frac{1}{N}\sum_{i=1}^{N}{\left(y_{i}-p_{i}\right)}^{2}\right]$$ (2)

Where $y_{i}$ represents the ground truth label of the i-th sample, $p_{i}$ represents the predicted probability of the i-th sample, and N represents the total number of samples.

### 2.4 Evaluation metrics

In this study, the prediction performance of DualPlaqueNet was compared with that of U-Net, ResUnet, and TransUNet. For the segmentation of carotid ultrasound images, the plaque region is considered the positive sample, while the non-plaque region is treated as the negative sample. These are categorized as true positives (TP), false positives (FP), true negatives (TN), and false negatives (FN). In this study, Accuracy, Mean Intersection over Union (MIoU), and Dice Similarity Coefficient (DSC) are used as evaluation metrics. Accuracy (ACC) reflects the ratio of correctly predicted pixels to the total number of pixels, with higher values indicating more precise segmentation. The Dice coefficient quantifies the similarity between the model’s predictions and the ground truth annotations. We have introduced the “background excluded mIoU” calculation method, which excludes background pixels (with a value of 0) in the mIoU calculation and only considers the IoU of the plaque area. This method avoids the influence of background areas on the evaluation results and more accurately reflects the segmentation performance of the model in patch areas. MIoU provides a more comprehensive evaluation of the model’s performance by averaging the IoU values for each class. The calculation Equations 3–5 for each evaluation metric are as follows:

$$Accuracy=\frac{TP+TN}{TP+TN+FP+FN}\times 100\%$$ (3)

$$MIoU=\frac{1}{k}\sum_{i=0}^{k}\frac{{TP}_{i}}{{FN}_{i}+{FP}_{i}+{TP}_{i}}$$ (4)

$$Dice=\frac{2TP}{2TP+FP+FN}$$ (5)

Among these, TP, FP, TN, FN, and k represent true positive, false positive, true negative, false negative, and the number of classes, respectively.

For the prediction of plaque size in carotid ultrasound images, this study employs the following three statistical metrics to evaluate the predictive performance on the test set. Mean Squared Error (MSE) is the mean of the squared differences between the predicted and actual values, while Mean Absolute Error (MAE) is the average of the absolute differences between the predicted and actual values. The smaller the MSE and MAE, the more accurate the predictions; *R* ^2^ measures the model’s ability to explain the variability of the data, and the closer *R* ^2^ is to 1, the stronger the model’s predictive performance. The calculation Equations 6–8 for these three statistical metrics are as follows:

$$MSE=\frac{1}{n}\sum_{i=1}^{n}{\left(y_{i}-{\hat{y}}_{i}\right)}^{2}$$ (6)

$$MAE=\frac{1}{n}\sum_{i=1}^{n}\left|y_{i}-{\hat{y}}_{i}\right|$$ (7)

$$R^{2}=1-\frac{\sum_{i=1}^{n}{\left(y_{i}-{\hat{y}}_{i}\right)}^{2}}{\sum_{i=1}^{n}{\left(y_{i}-\bar{y}\right)}^{2}}$$ (8)

Here, n represents the number of samples, $y_{i}$ denotes the actual values, ${\hat{y}}_{i}$ denotes the predicted values, and $\bar{y}$ represents the mean of the actual values.

## 3 Experiments and results

### 3.1 Experimental environment

The models in this study were implemented using Python 3.12.6 and PyTorch 2.4.1, and trained on an NVIDIA RTX 4060 GPU. The Adam optimizer (Aamir et al., 2023; Abirami et al., 2025) was used with an initial learning rate of 0.001. All models were trained with a batch size of 16 for 100 epochs.

### 3.2 Image segmentation

**FIGURE 3:**
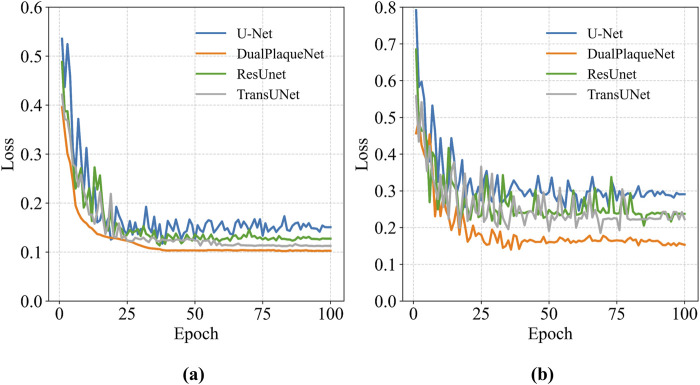
Loss curves of each model on the training set and validation set. **(a)** The loss curve of the training set, **(b)** The loss curve of the validation set.

After image preprocessing, the DualPlaqueNet model was trained on the data-augmented training set, while three other models were simultaneously trained for comparison (Figure 3). The validation set was used to tune hyperparameters and prevent overfitting during the training process. The test set was used to evaluate model performance on the region segmentation task and generate automatic segmentation result images of target regions.

We performed 10 repeated training sessions for DualPlaqueNet, U-Net, ResUnet, and TransUNet. After each training session, the optimal network parameters were saved, and then the average values and corresponding standard deviations of evaluation metrics for the 4 networks on the same test set were calculated. These results are shown in Table 3. The reason for conducting 10 repeated training sessions was primarily to evaluate the model’s stability and generalization ability, reducing the impact of random factors (such as parameter initialization and data order) during the training process on the final results. Due to these random factors, each training session may lead to different training results. Through multiple repeated training sessions and saving the optimal network parameters that performed best on the validation set in each training session, we were able to calculate the average performance and standard deviation of the model across multiple training sessions, thereby more reliably evaluating the model’s overall performance.

**TABLE 3:** Comparison of evaluation metrics between DualPlaqueNet and other network models on the same test set.

Method	MIoU (%)	IoU (excluding background) (%)	DSC (%)	ACC (%)
U-Net	84.19 ± 1.173	83.77 ± 1.168	85.46 ± 1.331	91.76 ± 0.139
ResUnet	85.27 ± 1.836	84.92 ± 1.215	86.13 ± 0.997	93.02 ± 0.226
TransUNet	86.42 ± 1.135	86.01 ± 1.098	86.79 ± 1.276	93.25 ± 0.352
DualPlaqueNet	88.91 ± 1.027	88.22 ± 1.065	89.95 ± 1.102	95.98 ± 0.073

IoU (excluding background): MIoU with background excluded, i.e., background pixels (value of 0) are excluded from MIoU calculation, considering only the IoU of plaque regions.

**FIGURE 4:**
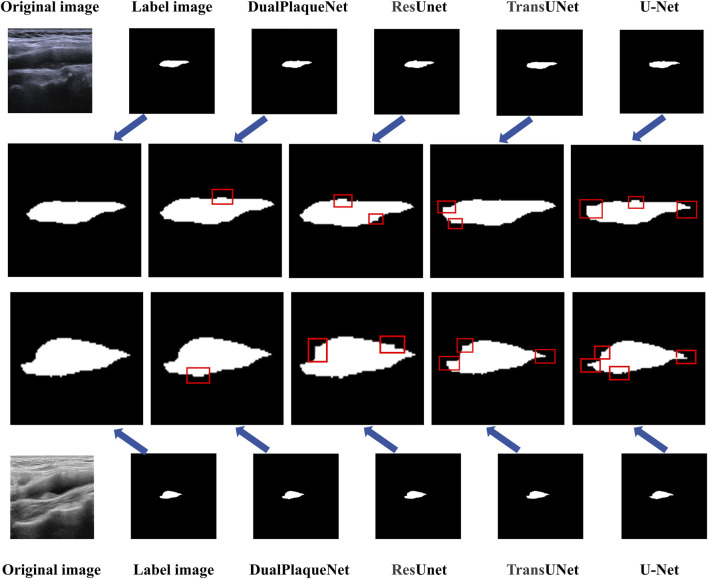
Comparison of image segmentation results from models.

Regarding the overfitting issue, we used the validation set during the training process to select optimal parameters and ensured that the final performance evaluation was conducted on the test set to validate the model’s generalization ability. Saving optimal network parameters does not mean the model has overfitted, because these optimal parameters were selected based on performance on the validation set, rather than solely relying on performance on the training set. This approach better ensures the model’s generalization ability and stability. Additionally, we also used techniques such as early stopping during the experimental process to prevent model overfitting, further ensuring that overfitting would not occur during the training process. Compared with the other three network models, DualPlaqueNet’s segmentation results were highly similar to doctors’ manual labels (Figure 4). This figure demonstrates that DualPlaqueNet is more sensitive to boundary information and closer to the true label images.

### 3.3 Plaque size prediction

For plaque size prediction, the training procedure is identical to that of image segmentation, using the augmented training set. During the manual annotation process by ultrasound physicians, the manually measured long and short diameters of the plaques were recorded in an Excel sheet and embedded into the metadata of the corresponding image files (written into DICOM private tags). In this study, DualPlaqueNet directly predicts the long and short diameters of the plaques, whereas U-Net first segments the images and then measures the segmented regions to obtain the long and short diameters. We conducted 10 repeated training sessions for both DualPlaqueNet and U-Net, saving the optimal network parameters after each training session. The average values and corresponding standard deviations of MSE, MAE, and *R* ^2^ on the same test set were calculated; these results are presented in Table 4. DualPlaqueNet achieved lower average MSE and MAE values and a higher average *R* ^2^ value compared to U-Net, indicating that DualPlaqueNet has a superior capability for predicting plaque size.

**TABLE 4:** Comparison of plaque size prediction performance between DualPlaqueNet and U-Net.

Evaluation indicators	DualPlaqueNet		U-net	
	Long diameter	Short diameter	Long diameter	Short diameter
MSE/mm^2^	1.08 ± 0.236	0.12 ± 0.047	1.53 ± 0.412	0.28 ± 0.072
MAE/mm	0.85 ± 0.174	0.28 ± 0.055	0.95 ± 0.304	0.43 ± 0.091
*R* ^2^	0.84 ± 0.038	0.79 ± 0.043	0.68 ± 0.088	0.55 ± 0.076

## 4 Discussion

In this study, a DualPlaqueNet model based on a multi-task joint learning framework was developed and validated, aiming to simultaneously achieve semantic segmentation and size prediction of carotid plaques. Experimental results show that, compared with the U-Net, ResUnet, and TransUNet models, DualPlaqueNet achieved significant advantages in MIoU, IoU, DSC, and ACC metrics. In predicting the plaque’s long and short diameters, its mean squared error and mean absolute error were both significantly reduced relative to U-Net, and the *R* ^2^ value also indicated a higher degree of fit. In this study, we adopted and improved the ECA (Efficient Channel Attention) module to enhance the performance of the model in plaque region segmentation. The design principle of the ECA module is to generate channel weights through local cross channel interactions, reducing computational overhead and achieving higher efficiency. The original ECA module used a layer of one-dimensional convolution to calculate channel weights. In this study, we introduced a multi-layer one-dimensional convolution structure inside the ECA module, which further optimized the calculation process of channel weights by extracting different levels of feature information layer by layer, enhancing the ability to capture complex image features. And, we will add it to the feature extraction section of the encoder. This design approach effectively overcomes the inherent limitations of ultrasound images, such as low contrast, high noise levels, and blurred edges, and significantly improves the model’s sensitivity to subtle changes in plaque characteristics, thereby maintaining high robustness and accuracy even in complex imaging backgrounds.

Currently, both domestic and international scholars have conducted extensive exploration and research in the field of carotid plaque detection and other medical image segmentation tasks (Chen et al., 2021; Flannery et al., 2021; Wang et al., 2021). Traditional image processing-based algorithms often focus on methods such as edge detection, which are limited by their sensitivity to noise and difficulty in characterizing complex lesion areas (Tsantis et al., 2014; Alshayeji et al., 2017; Zheng et al., 2015). In recent years, the introduction of deep learning technologies, such as CNNs, has provided a new breakthrough for addressing segmentation challenges in ultrasound and other medical images. For example, Yanhan Li et al. (Li et al., 2022) proposed a novel deep convolutional neural network model, FRDD-Net, for the automatic segmentation of carotid plaque ultrasound images. By incorporating a feature remapping module and a dense decoding mechanism, this model enhances feature extraction and utilization efficiency, overcoming the limitations of existing methods when dealing with low-quality images and irregular plaques. Experimental results indicate that FRDD-Net performs excellently on multiple datasets, demonstrating its potential and robustness in medical image segmentation tasks. Avesta A et al. (Avesta et al., 2023) proposed a brain image segmentation method based on a 3D Capsule Network (CapsNet), and compared it with traditional U-Net and nnUNet models. The experimental results show that CapsNet demonstrates significant advantages when processing the test set. Its segmentation accuracy is significantly higher than that of U-Net, and there is also a significant improvement in computational efficiency. CapsNet not only effectively segments brain structures but also requires lower memory and trains faster. Dong P et al. (Dong et al., 2024) introduced a UNet++ model enhanced with a dual-path attention mechanism (DPAM-UNet++) for the automatic segmentation of thyroid nodule ultrasound images. By integrating a dual-path attention module into the skip connections of UNet++, the model is able to effectively capture global contextual information, thereby improving the segmentation performance for small nodules and multiple nodules. Experimental results indicate that DPAM-UNet++ outperforms traditional segmentation models across multiple performance metrics, particularly in enhancing boundary precision and handling multiple nodules. Compared to the aforementioned works, this study leverages the advantages of traditional deep learning frameworks while organically integrating plaque semantic segmentation and size prediction through a multi-task joint optimization strategy. This approach enables comprehensive information sharing and complementarity, helping to overcome the limitations of single-task methods in information extraction, thereby providing a more comprehensive and efficient technical means for the quantitative analysis of carotid plaques.

The DualPlaqueNet model presented in this study embodies both foresight and practical value in its design. By introducing a dual-branch structure and a cross-fusion strategy, the model achieves collaborative learning of plaque morphology and size information. This multi-task joint learning approach overcomes the limitations of previous single-objective optimizations, effectively enhancing the model’s performance in the complex environments of ultrasound imaging. Additionally, the embedded attention mechanism allows the model to automatically focus on key feature regions, further improving the extraction of both global semantic information and local detail features, thereby optimizing plaque region segmentation and size prediction. Nevertheless, there are certain limitations to this approach. First, the model’s training and validation were conducted on a single-center dataset with a relatively limited amount of data, which might lead to insufficient generalization performance in multi-center or multi-device application scenarios. Second, the inherent noise and variability in ultrasound images can result in local misjudgments, especially in regions with fuzzy edges or low contrast. Moreover, although multi-task learning facilitates feature sharing to a certain extent, the challenge of balancing the different tasks still requires further investigation. How to adaptively adjust task weights under varying data distributions remains a direction for future research. In summary, DualPlaqueNet shows significant advantages in improving automated plaque detection and quantitative analysis, offering considerable support to ultrasound physicians and enhancing the diagnostic efficiency for carotid plaques. However, for its practical application and broader clinical promotion, continuous optimization is necessary. This includes increasing sample sizes, incorporating multi-center data, and further refining the model architecture to ensure stable and efficient performance in a wider range of clinical scenarios.

## 5 Conclusion

This study proposes the DualPlaqueNet model, which integrates a dual-branch structure and attention mechanism. Through comparisons with models such as U-Net, ResUnet, and TransUNet, it was found that DualPlaqueNet demonstrates excellent performance in both semantic segmentation and size prediction tasks for carotid artery plaques, showing promise as a tool to assist in early screening and risk assessment of cerebrovascular diseases.

## Data Availability

The raw data supporting the conclusions of this article will be made available by the authors, without undue reservation.
